# The Association of Hematological Malignancy and End-of-Life Expenditure in Cancer Decedents: A Population-Based Study in an Asian Country

**DOI:** 10.1097/MD.0000000000001036

**Published:** 2015-07-02

**Authors:** Yeh-Ting Hung, Chih-Yuan Huang, Chun-Ming Chang, Ching-Chih Lee

**Affiliations:** From the Department of Pediatrics, Dalin Tzu Chi Hospital, Buddhist Tzu Chi Medical Foundation, Chiayi, Taiwan (Y-TH); Division of Nephrology, Department of Internal Medicine, Ditmanson Medical Foundation Chia-Yi Christian Hospital, Chia-Yi City, Taiwan (C-YH); Department of Otolaryngology, Dalin Tzu Chi Hospital, Buddhist Tzu Chi Medical Foundation, Chiayi, Taiwan (C-CL); Center for Clinical Epidemiology and Biostatistics, Dalin Tzu Chi Hospital, Buddhist Tzu Chi Medical Foundation, Chiayi, Taiwan (C-CL); School of Medicine, Tzu Chi University, Hualian, Taiwan (Y-TH, C-MC, C-CL); Community Medicine Research Center and the Institute of Public Health, National Yang-Ming University, Taipei, Taiwan (C-CL); and Department of Surgery, Dalin Tzu Chi Hospital, Buddhist Tzu Chi Medical Foundation, Chiayi, Taiwan (C-MC).

## Abstract

Within the overall National Health Insurance (NHI) budget in Taiwan, there has been a remarkable increase in expenditure for cancer patients. This study was designed to explore whether hematological malignancy is associated with higher end-of-life (EOL) medical expenditure in their last 6 months of life.

We used data from the Taiwan NHI Research Database to do a retrospective cohort and population-based study. There were 42,754 cancer patients enrolled in order to study the determinants of medical expenditure for EOL care from 2009 to 2011.

The mean medical expenditure for EOL care for cancer patients in the last 6 months of life was $12,965 ± 10,959 (mean ± standard deviation ) (all costs are given in US dollars). Patients with acute leukemia and lymphoma had an additional cost of $16,934 and $7840 than those with nonhematological malignancy (*P* < 0.001). Medical expenditures for cancer patients with a hematological malignancy and postdiagnosis survival of >6 months, between 6 and 12 months, and >12 months all showed that acute leukemia and lymphoma accounted for more medical expenditure than did others (*P* < 0.001). The primary physician's specialty between acute leukemia, lymphoma, and nonhematological malignancy patients had statistically difference.

The medical expenditure of cancer patients in acute leukemia and lymphoma was more than nonhematological malignancy. Treatment strategies for acute leukemia should be studied further in order to save the health care budget.

## INTRODUCTION

End-of-life (EOL) care has consumed a significant proportion of health care resources and become aggressive over the past decade, resulting in disproportionately greater medical expenditures for decedents than for survivors.^1,2^ Fassbender et al^2^ found that decedents made up 1.1% of the population but consumed 21.3% of health care costs in the final 6 months of life, and concluded that diagnoses of illnesses responsible for death are significant determinants of functional decline over time and identified that the latest year costs were high for organ failure, terminal illness, and frailty, and lowest for the sudden death category.

The estimated death rates for males and females due to leukemia and non-Hodgkin lymphoma were within the top 10 fatal cancers in the United States in 2010.^3^ For cancer decedents, patients with hematological malignancy represented a special group. Howell et al^4^ showed that patients with hematological malignancies were less likely to receive hospice services compared to other cancers and might be due to ongoing management by the hematology team and consequent strong bonds between staff and patients; uncertain transitions to a palliative approach to care; and sudden transitions, leaving little time for palliative input. In Australia, McGrath et al^5^. pointed out the benefits of palliative care at EOL care, but, for many hematology patients, there are still problems with timely referrals to the palliative care system. This phenomenon raised a new issue whether cancer decedents with hematological malignancy incurred higher EOL expenditure.

The aim of this study was to analyze the medical expenditure from the National Health Insurance (NHI) Research Database for cancer decedents in order to explore whether hematological expenditure is associated with higher EOL medical expenditure in their last 6 months of life. This information may provide us more information about the EOL expenditure among cancer patients and may offer a chance to change public health strategy to decrease economic burden for health care system.

## PATIENTS AND METHODS

### Ethics Statement

This study was approved by the Institutional Review Board of Buddhist Dalin Tzu Chi General Hospital, Taiwan. The review board stated that written consent from patients was not required because the identification numbers and personal information about individuals included in the study were not included in the secondary files.

### Patients and Study Design

We linked individual patient-level data with encrypted personal identification numbers from computerized data from the National Register of Deaths Database and NHI Research Database for our retrospective cohort study. The government was the only insurer in this single-payer system and provided comprehensive services and universal insurance coverage. Patients were free to choose any health care provider. Taiwan's Department of Health monitored the databases for accuracy. From 2009 to 2011, a total of 78,613 cancer deaths were identified in the National Register of Deaths Database. We collected data from NHI databases of inpatient or outpatient claims and identified 42,754 primary cancer patients without metastasis with a postdiagnosis survival time of >6 months. There were 100 hospitals included in the analysis. Among them, we compared the medical expenditures for 275 acute leukemia patients (acute myeloid leukemia [AML] and acute lymphoblastic leukemia [ALL]) and 704 lymphoma patients (Hodgkin lymphoma and non-Hodgkin lymphoma) with 41,775 nonhematological malignancies patients. We also compared the characteristics for cancer patients in terms of socioeconomic status (SES), hospital spending index, sex, age, severity of disease, postdiagnosis survival time, primary physician's specialty, hospital characteristics, caseload group, urbanization level of residence, geographic location, and the year in which medical expenditures were recorded.

### Patient Demographics

Patient characteristics included age group (18–34.99, 35–44.99, 45–54.99, 55–64.99, 65–74.99, 75–84.99, and older than 85), sex, hospital spending index, cancer diagnosis, postdiagnosis survival time, Charlson Comorbidity Index Score (CCIS), primary physician's specialty, hospital characteristics, caseload group, urbanization level of residence, geographic location, year in which medical expenditures were recorded, and SES. We used the Deyo adaptation of the CCIS which was derived from inpatient diagnosis in the last 6 months of life to estimate disease severity.^6^ We found the primary physician's specialty from NHI claims and these were dichotomized into oncologist and other. We identified comorbid conditions from ICD-9 codes in NHI claims for both inpatients and outpatients in the last 6 months before death. We used enrollee category (EC) to measure SES because SES is an important prognostic factor in cancer.^7,8^ We classified the cancer patients into 3 subgroups: high SES (full-time or regular paid personnel with a government affiliation, civil servants, or employees of privately owned institutions), moderate SES (members of the farmers’ or fishermen's associations, self-employed individuals, and other employees), and low SES (substitute service draftees, low-income families, and veterans).^9^

### Statistical Analysis

We used the SPSS (version 15; SPSS Inc, Chicago, IL) to analyze data. We used one-way ANOVA test for continuous variables, the χ^2^ test for categorical variables, and compared characteristics of patients and the care they received by univariate analyses in Table 1 . We used multilevel analysis using hospital as a random-intercept model to analyze the effect of each explanatory variable on the medical expenditure for EOL care. This meant a mixed-effects model with hospital as a random effect, and the patient characteristics as fixed effects. A mixed model is a statistical model containing both fixed effects and random effects, that is mixed effects. In Table 2, we used multivariate analysis using a random-intercept model to compare the EOL medical expenditure in their last 6 months of life between acute leukemia and nonhematological malignancy, and between lymphoma and nonhematological malignancy. In Table 3, we used multivariate analysis using a random-intercept model to compare the EOL medical expenditure in their last 6 months of life between acute leukemia and nonhematological malignancy, and between lymphoma and nonhematological malignancy who survived 6.01–12 months and >12.01 months. We used a 2-tailed value of *P* < 0.05 to determine statistical significance.

**TABLE 1:**
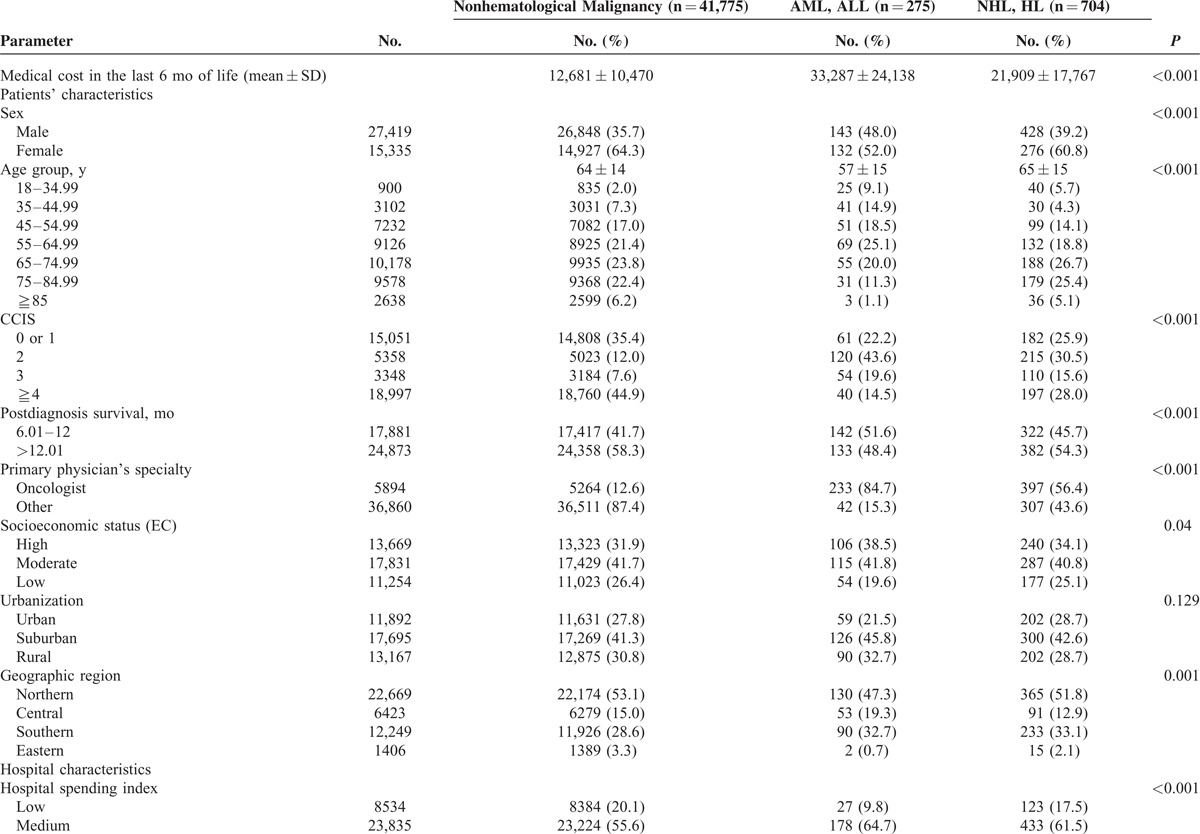
Baseline Characteristics Hematological Malignancy Decedents From 2009 to 2011(n = 42,754)

**TABLE 1 (Continued):**
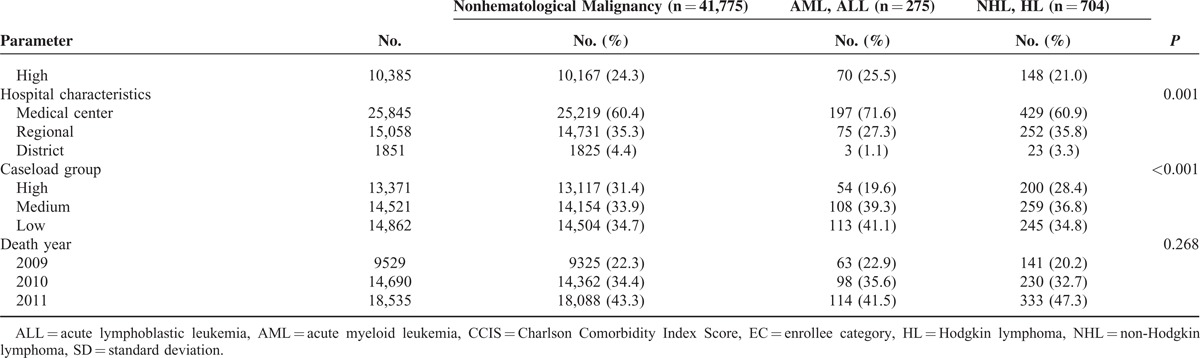
Baseline Characteristics Hematological Malignancy Decedents From 2009 to 2011(n = 42,754)

**TABLE 2:**
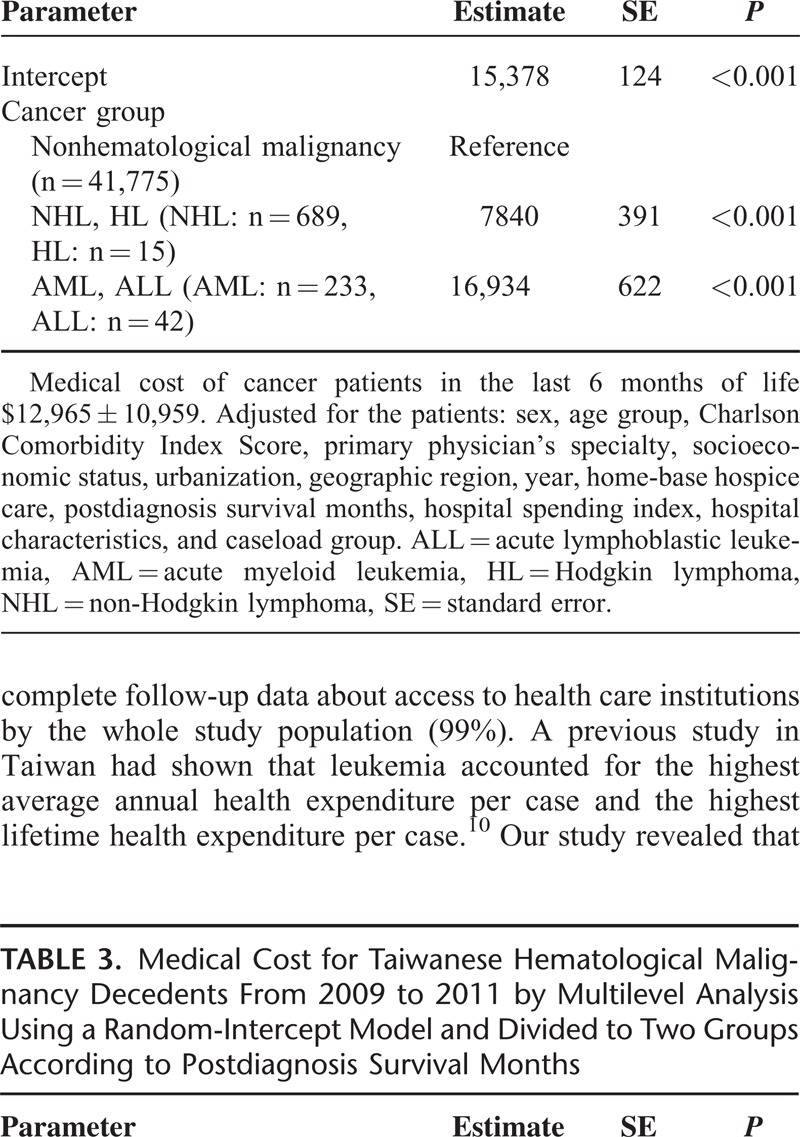
Medical Cost for Taiwanese Hematological Malignancy Decedents From 2009 to 2011 by Multivariate Analysis Using a Random-Intercept Model (n = 42,754)

**TABLE 3:**
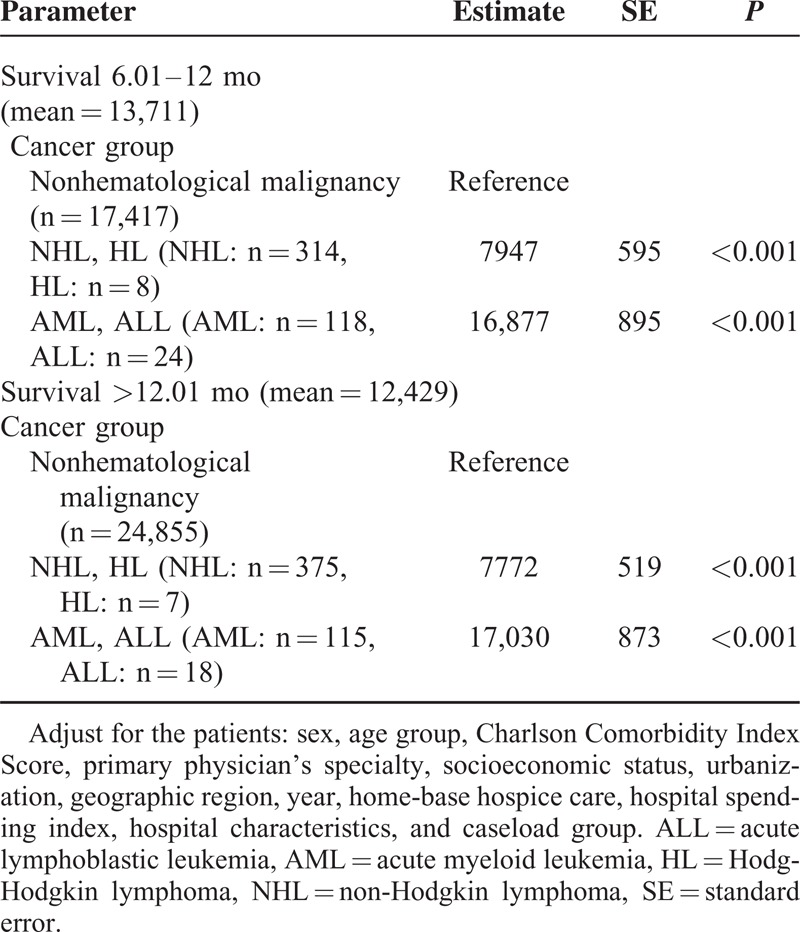
Medical Cost for Taiwanese Hematological Malignancy Decedents From 2009 to 2011 by Multilevel Analysis Using a Random-Intercept Model and Divided to Two Groups According to Postdiagnosis Survival Months

## RESULTS

A total of 42,754 cancer decedents from 2009 to 2011 were included in this study. Their basic characteristics and medical expenditures for EOL treatments in the last 6 months of life are shown in Table 1 . The age and primary physician's specialty between acute leukemia, lymphoma, and nonhematological malignancy patients had statistically difference. Patients with acute leukemia and lymphoma who had CCIS 2 had more patient numbers than others. Female sex, age >45 years old, medical center hospital, moderate SES, and hospital with a medium spending index were associated with more patient numbers in cancer patients.

The medical cost in the last 6 months of life in cancer patients with acute leukemia and lymphoma had an additional cost of $16,934 and $7840 than those with nonhematological malignancy (*P* < 0.001) (Table 2). Figure 1 depicts the medical expenditure in the last 6 months of life in cancer patients, and Figure 2A for those with postdiagnosis survival between 6 and 12 months and Figure 2B for those with postdiagnosis survival of >12 months. Medical expenditures of last 6 months of life for cancer patients with a hematological malignancy and postdiagnosis survival of >6 months, between 6 and 12 months, and >12 months all showed that acute leukemia and lymphoma accounted for more medical expenditure than did others (*P* < 0.001) (Tables 2 and 3) (Figures 1 and 2A and B).

**FIGURE 1:**
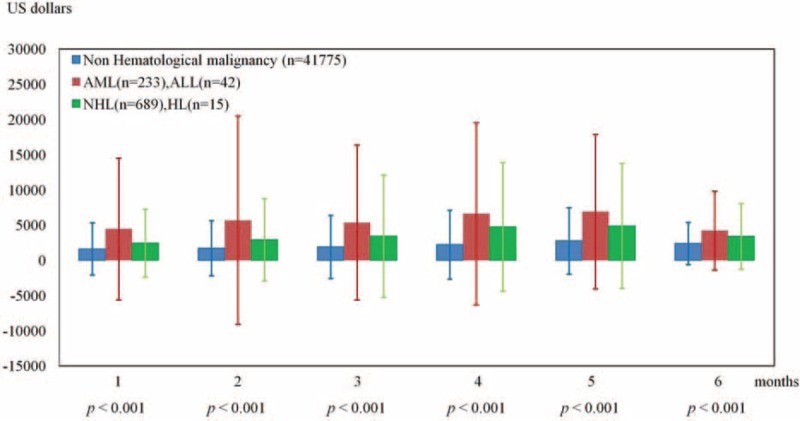
Medical expenditure in the last 6 months of life in cancer patients.

**FIGURE 2:**
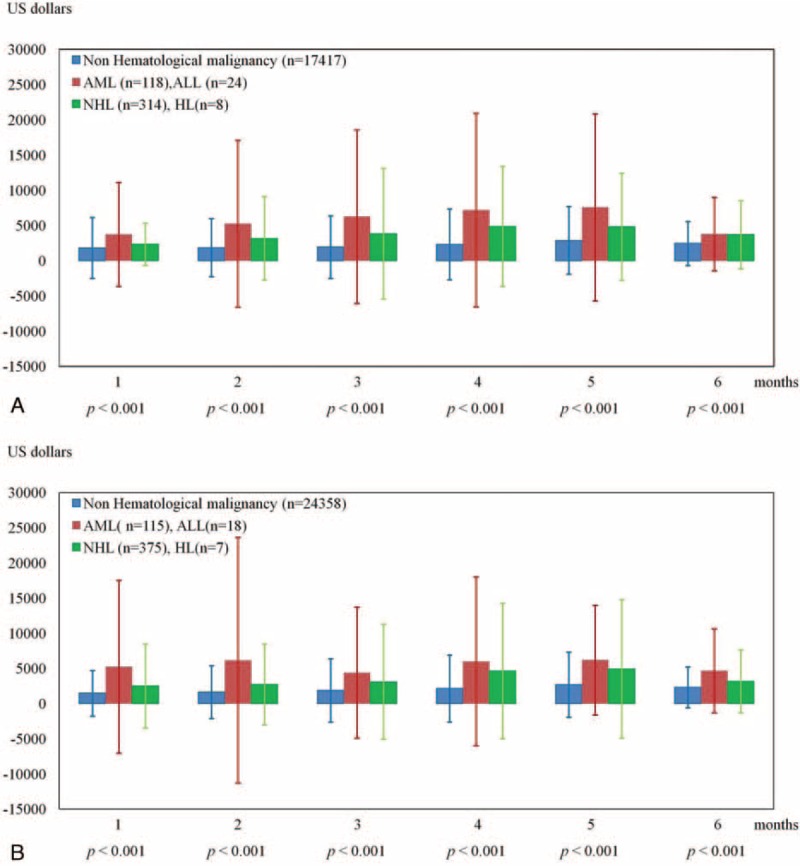
(A) Medical expenditure in the last 6 months of life in cancer patients with postdiagnosis survival between 6 and 12 months. (B) Medical expenditure in the last 6 months of life in cancer patients with postdiagnosis survival >12 months.

In hierarchical linear modeling analysis, patients with acute leukemia incurred an additional 131% EOL expenditure, compared with nonhematological malignancy. The mean EOL expenditure for the last 6 months of life in cancer patients were $12,965 ± 10,959 (mean ± standard deviation) was shown in Table 2.

We further stratify our data into 2 groups according to the postdiagnosis survival because the postdiagnosis survival is strongly associated with the EOL expenditure shown in Table 3. Medical expenditures for cancer patients with a hematological malignancy and postdiagnosis survival between 6 and 12 months and >12 months all showed that acute leukemia and lymphoma accounted for more medical expenditure than did others (*P* < 0.001) (Table 3).

## DISCUSSION

In this national database study of cancer decedents from 2009 to 2011, we found that acute leukemia and lymphoma patients accounted for greater EOL medical expenditure than did those with other cancers. These results were robust even we stratified the patients by different postdiagnosis survival time.

Our study was based on a nationwide population-based cross-sectional study, the diagnostic accuracy of which was monitored by the NHI Bureau of Taiwan and included almost complete follow-up data about access to health care institutions by the whole study population (99%). A previous study in Taiwan had shown that leukemia accounted for the highest average annual health expenditure per case and the highest lifetime health expenditure per case.^10^ Our study revealed that acute leukemia and lymphoma patients accounted for more EOL medical costs than other cancer patients in Taiwan.

Cancer results in a high economic burden.^11^ Our study showed that medical expenditure was higher for acute leukemia and lymphoma patients than for other cancer patients. This may have been because patients with hematological malignancies were less likely to receive care from palliative specialists or hospice services when compared to patients with other cancers.^4,12^ The incidence of AML among the elderly grows as the population continues to age and most older patients do not receive hospice services.^13^ Nordmann et al^14^ found that the chief costs associated with AML were therapy and personnel costs for nursing staff, followed by hotel business and personnel costs for doctors and diagnostic procedures. The high cost of lymphoma might be related to its high prevalence among the elderly as life expectancy increases, and Foster et al identified the direct cost of chemotherapy as the most common factor in Follicular non-Hodgkin lymphoma.^11,15^ Although all treatment costs for AML patients increased, the effectiveness of these treatments also increased, resulting in an increased rate and duration of survival, and several patients were long-term survivors.^14,16^ Acute leukemia and lymphoma decedents in the last 6 months of life accounted for more medical expenditures than other cancer decedents might have been because they had complications during treatment such as febrile neutropenia, infection, graft-versus-host disease, and transplantation for more advanced disease.^13,15,17,18^ In Taiwan, the aggressiveness of EOL care has been noted for years.^19^ If health care professionals informed terminally ill cancer patients about their prognosis and discussed the goals of future care, then they might not make overly aggressive treatment decisions.^20,21^ To save on medical expenditure for acute leukemia and lymphoma cancer decedents, the authorities might make an effort to encourage early discussion with terminally ill cancer patients and change aggressive EOL treatment to hospice care. These study results might help in that regard.

There are some limitations to this study. The cancer diagnoses and comorbidities were dependent on ICD codes; however, the charts and patient interviews were reviewed randomly by the NHI Bureau of Taiwan to confirm the diagnoses. Besides, cancer decedents could not be thought terminally ill and our study did not bring up health care qualities. The information from the NHI Bureau of Taiwan also lack cancer stage, disease severity, and pathology report; thus, we could not analyze whether those factors were associated with medical expenditure. Future research should investigate the factors associated with late transfer to palliative care and the determinants of medical expenditures for acute leukemia.
